# Superhydrophobic Surface Preparation and Wettability Transition of Titanium Alloy with Micro/Nano Hierarchical Texture

**DOI:** 10.3390/ma11112210

**Published:** 2018-11-07

**Authors:** Zhiru Yang, Chongchong Zhu, Nan Zheng, Dezheng Le, Jianzhong Zhou

**Affiliations:** School of Mechanical Engineering, Jiangsu University, Zhenjiang 212013, Jiangsu, China; wan_009@126.com (C.Z.); zn-andre@hotmail.com (N.Z.); xxzj_003@126.com (D.L.); zhoujz@ujs.edu.cn (J.Z.)

**Keywords:** laser surface texturing, hierarchical structure, low-temperature annealing, wettability transition, surface chemistry

## Abstract

Microstructures are applied to various hydrophobic/hydrophilic surfaces due to the role of adjusting the surface wettability. In this paper, a 1064 nm pulsed picosecond laser was applied to prepare a micro/nano hierarchical structure on the surface of the titanium alloy (Ti-6Al-4V). The microstructures consist of dimple arrays with various diameters, depths, and areal densities. They are obtained by controlling the pulse energy and the number of pulses. The nanostructures are periodic ripples, which are defined as laser-induced periodic surface structure (LIPSS), and the dimensional parameter of LIPSS can be adjusted by changing the laser energy density and scanning speed. The contact angles of various laser textured surfaces were measured. It is found that the contact angle increases with the density of micro-textured surface increases, and the wetting state of textured surfaces conforms to the Cassie model. Some laser processed samples were subjected to low-temperature annealing treatment. It is observed that the low-temperature annealing process can accelerate the surface wettability transition significantly, which is attributed to the change of the hydroxyl groups on the surface. Finally, a superhydrophobic surface with the maximum contact angle of 144.58° is obtained.

## 1. Introduction

The wettability transition of hydrophobicity/hydrophilicity surfaces has become a hot topic in recent years. Surfaces of animals and plants have microstructures, such as lotus leaves or shark skin. They exhibit unique wetting properties such as superhydrophobicity and self-cleaning ability [1], antifouling and anti-biofouling [2], etc. Wetting properties of surfaces are affected by the micron and nanoscale hierarchical structure as well as the surface chemistry synergistically [3].

**Figure 1:**
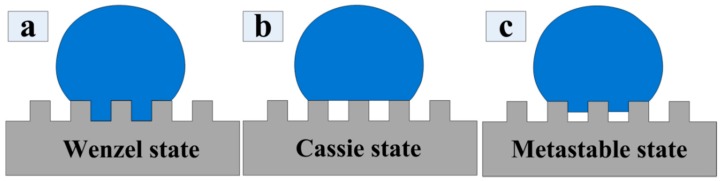
Schematic diagrams of the Wenzel state, Cassie state, and metastable wetting state.

The wettability of the surface is divided into hydrophobic and hydrophilic properties, which are studied by the surface contact angle (CA). That is, the surface with a CA higher than 90° is a hydrophobic surface. Furthermore, the surface that satisfies the condition of a CA above 150° and a rolling angle of less than 5° is called a superhydrophobic surface. The infiltration state of a water droplet on the solid surface is generally divided into three categories, the Wenzel state, the Cassie–Baxter state, and the metastable state [4,5]. The diagram of the infiltration state is shown in Figure 1. Among those wetting states, the Cassie–Baxter state is more suitable for the study of the hydrophobic textured surface [6].

Hydrophobic surfaces have advantages in corrosion resistance [7,8], biocompatibility [9], and extreme environments (ocean, dust) [10,11]. Surface microtexture preparation and low surface energy modification, are the two aspects to improve surface hydrophobicity. There are many methods to fabricate micro-texture and get superhydrophobic surfaces by changing the surface morphological structures, such as shot blasting [12], chemical etching [13], machining [14], plasma etching [15], etc. However, the techniques above have some shortcomings that limit the application. For example, the complicated steps, harsh service environment, and low service life. Besides, the surface modification is applied to reduce the free energy of a surface coated with some coatings, such as fluorosilane (PFOS) and fluoropolymer (CHF3 or C4F8) and so on. Then, the hydrophobicity of the surface can be significantly improved [16,17,18]. However, these organic compound coatings may be peeled off from the substrate due to low mechanical and thermal stability. Moreover, these compounds are toxic, which may result in poor compatibility of the surface with the biological environment, and limit the application of hydrophobic surfaces.

Compared with the above processing technology, laser surface texturing technology [19,20,21,22] can realize the preparation of large-area functional surface through simple and rapid processing, and be applied to industrial production with high reproducibility. Ultrashort pulse laser, including femtosecond and picosecond lasers, have higher precision for preparation of micro/nano-textured surfaces particularly [21]. Recently, there have been studies that hydrophobic properties can also be obtained on the surface of materials only by laser surface texturing techniques. Ta et al. used a nanosecond fiber SPI laser to fabricate the grid structure at equal intervals on the surface of the 304S15 stainless steel. The test results showed that the surface was superhydrophobic and the contact angle reached 155° [23]. Picosecond laser was applied to prepare mesh structures of different sizes and different grooves on the surface of aluminum alloy by Li et al. The results showed that the obtained surface achieved self-cleaning ability and excellent hydrophobicity [24]. Li et al. used nanosecond laser technology to prepare linear patterns, grid patterns, and dimple array patterns on the surface of a Ti-6Al-4V substrate. The wettability transition was obtained without any modification of low surface energy and the maximum contact angle was 137° [25]. Yang et al. attempted to prepare patterns on the IN718 surface only by nanosecond laser processing. The contact angle test results showed that the surface obtained high hydrophobicity [26]. In addition, some researchers tried to prepare a biomimetic hierarchical structure on the surface of substrates [27,28,29,30], and the results revealed that the transformation from hydrophilic to hydrophobic was realized.

However, it has been found that the surface of the laser textured surface exhibits high hydrophilicity in the early stage of preparation. Then the contact angle increases with storage time and achieves stable hydrophobic properties. The periods of wettability transition in various substrates are different [31,32,33,34,35,36]. The reason for wettability transition has caught lots of attention [37,38,39,40,41,42]. Zhong et al. stored aluminum samples textured by picosecond laser in different conditions such as air, CO_2_, O_2_, and N_2_ atmospheres, and gas environments with the rich organic compound. The adsorption of organic compounds in the air plays a key role in this process. Higher C/Al and C-C(H) (at %) values indicate a more nonpolar surface which tends to be hydrophobic [29]. When the textured copper samples were stored in an organic-rich or vacuum environment, the CA reached 134.1° ± 3.3° and 121.1° ± 5.1° after eight days, respectively. The value of C/Cu increased from 0.74 to 1.30, and finally 1.60 [37]. The wettability transition time can be shortened. Yan et al. stored laser textured brass in different media and found that CA increased sharply from 12.6° to 116.9° after four days of air exposure. However, the surface became hydrophobic immediately by immersing in isopropanol (IPA) for 3 h. They informed that the surface hydrophobicity was related to the increase of surface carbon atom percentage (C = 58.59%) [38]. Lei et al. prepared a superhydrophobic silver surface on brass by electrodeposition. At first, the surface was subjected to heat treatment at 200 °C and then the contact angle test was conducted after cooling, and the surface was superhydrophobic immediately [39]. Qing et al. prepared the TiO_2_/PVDF superhydrophobic hierarchical surface. They found that the transition time was shortened from 200 min to less than 30 min when the heating temperature was 180° [40]. The study of Chun et al. demonstrated that low-temperature annealing (100 °C) contributed to accelerating the wettability transition of the nanosecond laser textured copper surface from hydrophily to superhydrophobicity. The transition time can be reduced from a few weeks to a few hours, while ethanol was also used as a better reductant [41]. Chun et al. studied the effect of low-temperature annealing on the surface hydrophobicity of stainless-steel textured nanosecond laser. The results showed that the transition time of the sample treated by low-temperature annealing was shortened from one month to four hours [42]. The low-temperature annealing process is a simple and effective method to accelerate the chemical conversion of the surface and the adsorption of organic matter. In summary, low-temperature annealing is an effective means of accelerating hydrophobic transitions.

In this paper, a micro/nano hierarchical structure with micro-dimple array and LIPSS was fabricated on the surface of Ti-6Al-4V by picosecond laser texturing. Based on the infiltration model (Cassie and Wenzel model), the effect of different structural shape and dimension on surface wettability was discussed. Moreover, the low-temperature annealing process was combined with energy dispersive spectrometer (EDS) to analyze and discuss the mechanism of surface wettability transition of micro-textured on the Ti-6Al-4V surface. Finally, a surface hydrophobicity regulation method was proposed.

## 2. Experimental Sections

### 2.1. Preparation of Materials

The Ti-6Al-4V titanium alloy sheet was prepared by a wire cutting process with a size of 20 × 20 × 4 mm. Then the surface was polished with metallography sandpaper of #180, #600, #1000, #1500, and #2000. Before and after being treated by the laser the samples were cleaned in a 10 min ultrasonic acetone bath, followed by a 10 min ethanol bath and dried in an oven for 30 min at the temperature of 100 °C.

### 2.2. Equipment and Process Parameters

**Figure 2:**
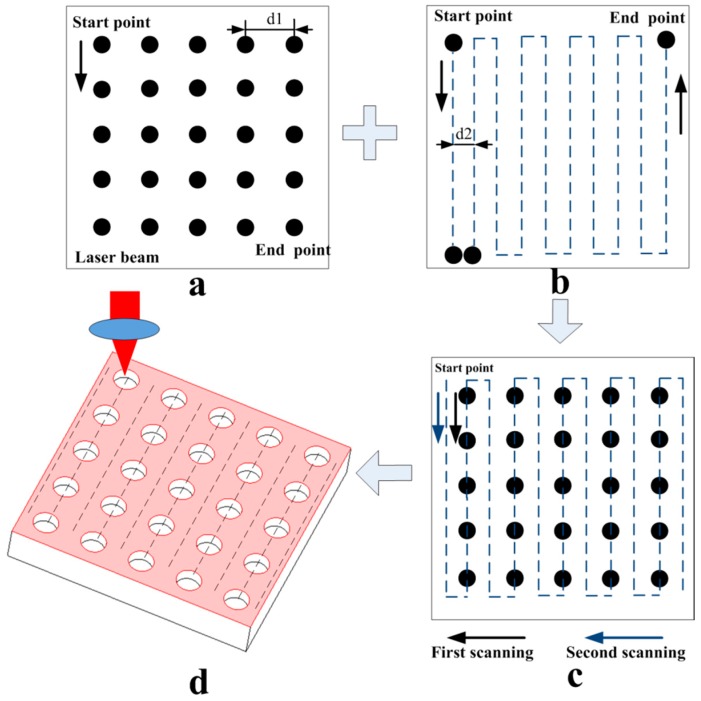
Schematic diagrams of the micro/nanostructure prepared by laser treatment on the Ti-6Al-4V surface. The scanning paths: (**a**) the micro-dimple array; (**b**) the LIPSS ripple; (**c**) hierarchical structure; (**d**) the 3D schematic diagram of a laser textured hierarchical structure.

The ultrashort pulse picosecond laser system (EdgeWave (PX100-1-GM), Delphilaser Inc., Suzhou, China) was applied to scan Ti-6Al-4V alloy in the air with a pulse width of 12 ps and laser wavelength of 1064 nm. The maximum power of the laser is 70 W and the maximum single pulse energy is 250 μJ. Online monitoring of structured surface morphology is carried on by combining the focus objective lens and the CCD camera (Delphilaser Inc., Suzhou, China). The laser source moves along the designed scanning path showed in Figure 2. A fast shutter opens for a short period of time with the sample stationery to control the pulse count and form spaced micro-dimples eventually. The spot diameter is approximately 20 μm, which could be controlled by adjusting the amount of defocusing with the range of 0~4 mm. Furthermore, the laser energy density is from 0.02 J/cm^2^ to 66.8 J/cm^2^. Table 1 shows the specific process parameters selected for the preparation of different structural features, respectively, including micro-dimple array and LIPSS.

### 2.3. Micro/Nanostructure Design

In this experiment, a micro/nano hierarchical structure was prepared by a two-step picosecond laser ablation process. The first step is to prepare a micro-dimple array by adjusting the pitch between micro-dimples with 100, 80, and 60 μm, which corresponding to different areal densities of 13, 20, and 35%, respectively. The aspect ratio is 50%. The main process parameters are shown in Table 1a. The second step is the preparation of a laser-induced periodic surface structure (LIPSS). The LIPSS was fabricated on the surface by laser-induced technique. The main process parameters are shown in Table 1b. Finally, the surface with the micro-dimple array structure was covered with the LIPSS uniformly. The texture area of different structural features is 0.8 × 0.8 mm^2^. A schematic diagram of a specific micro-texture model is shown in Figure 2.

### 2.4. Low-Temperature Annealing Treatment

In order to accelerate the wettability transition, some samples were subjected to low-temperature annealing treatment in an electric resistance furnace. The annealing temperature is 150 °C [39,40,41,42], and the annealing time is 500 min [42].

### 2.5. Characterization

The digital microscope was used to characterize the two-dimensional and three-dimensional images of the micro-dimple structure of the sample to determine the structural parameters including the diameter and depth of the micro-dimple. Besides, the morphology of three characteristic structures, including micro-dimple array, LIPSS, and micro/nano hierarchical structure were observed by 3400 N tungsten filament scanning electron microscope (SEM, Hitachi, Tokyo, Japan). The contact angle (CA) measurements were obtained at room temperature with an optical contact angle measuring instrument (Dataphysics, Stuttgart, Germany) based on the static sessile drop method. The volume of the water droplet was 5 μL. The CA was measured at three different spots on the same surface with an average error of ±5°. An energy dispersive X-ray spectroscope (EDX) was used to analyze and research the change of surface composition after laser surface texturing and low-temperature annealing treatment.

## 3. Results and Discussion

### 3.1. Micro-Dimple Array Fabrication

#### 3.1.1. Laser Parameters vs. Structure Parameters

The laser scans the same position at a certain interval [43], which can reduce heat accumulation and improve the texture quality. As the amount of defocus increases, the laser spot diameter increases. When the amount of defocus is between 0.5 mm and 2.5 mm, a micro-dimple of specific diameter can be obtained. As the laser spot diameter increases, the single point energy density is lowered. A micro-dimple of specific depth can be fabricated by increasing the number of cumulative pulses, and the number of pulses is between 20 and 200 in this experiment.

**Figure 3:**
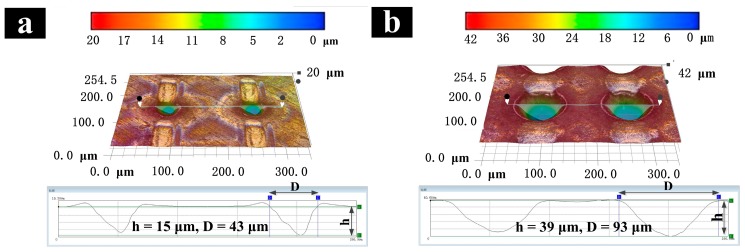
2D and 3D morphology of micro-dimple array different Laser parameters: (**a**) Defocus amount L = 0.5 mm, Pulse number N = 40; (**b**) L = 2 mm, N = 100.

The single micro-dimple morphology is shown in Figure 3. Figure 3a,b represent different micro-dimple obtained under two typical parameters, respectively. By comparing the 2D and 3D morphology of the micro-dimple, it can be observed that the smaller defocus amount corresponds to a small micro-dimple diameter, and the larger pulse number corresponds to a larger depth. In order to obtain micro-dimple of accurate size, it is necessary to study the effects of the pulse number and the defocus amount on the depth of the micro-dimple.

**Figure 4:**
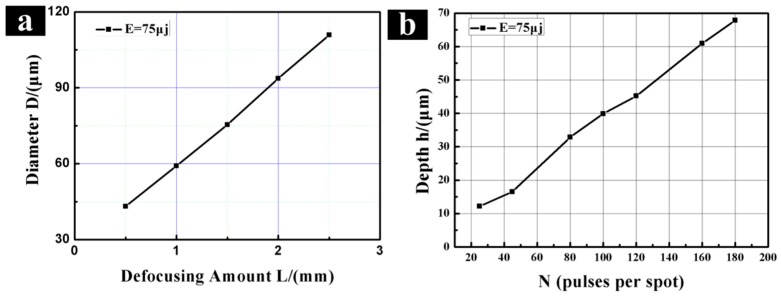
The relationship of the irradiation parameters with the desired geometry. (**a**) Diameter vs. the defocusing amount; (**b**) depth vs. the pulses per spot.

The relationship between the laser defocus amount and the micro-dimple diameter is studied, and the relationship between the pulse numbers and the micro-dimple depth is also studied. The results are shown in Figure 4. In Figure 4a, the dimple diameter increases linearly with the rises of defocus amount. When the defocus amount increases, the laser spot diameter increases. Then, the dimple diameter increases. The depth of the micro-dimple is related to the number pulses. Figure 4b shows the relationship between the depth of micro-dimples and pulses. The depth increases linearly with the increase of pulses. When pulses are 25, the depth is 12 μm. When the N increases to 180, the depth reaches a maximum value of 67 μm.

#### 3.1.2. Preparation of Micro-dimple Array Structure

**Figure 5:**
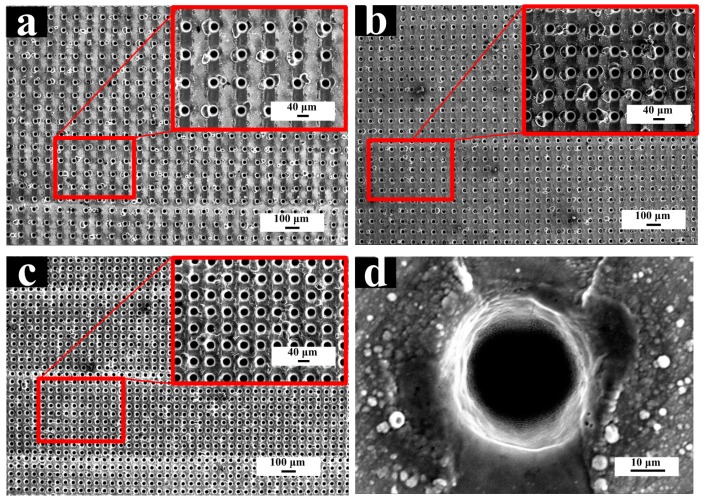
FE-SEM images of processed the micro-dimple array: (**a**) the pitch distance = 100 μm; (**b**) the pitch distance = 80 μm; (**c**) the pitch distance = 60 μm; (**d**) the morphology of a single micro-dimple.

The different areal densities of the micro-dimple array are prepared by adjusting the pitch of adjacent action spots, which is shown in Figure 5. The micro-dimple diameter is 40 μm, and the pitch is set to 100, 80, and 60 μm, which is corresponding to areal densities with 13, 20, and 35%, respectively. The micro-dimple array was observed and characterized by scanning electron microscopy and Figure 4 is a corresponding electron microscope image. It can be observed in Figure 4d that the surfaces of the micro-dimple and its peripheral regions are free of splashing of molten residue. Then the smooth and accurate micro-dimple is obtained, which meets the expected requirements.

### 3.2. Nanoscale Pattern Preparation

#### 3.2.1. Parameters of LIPSS

The prepared LIPSS in this experiment are periodic ripples. They are divided into HSFL (high spatial frequency LIPSS) and LSFL (low spatial frequency LIPSS) two types. The period of HSFL is less than half of the laser wavelength. Meanwhile, the period of LSFL is close to the laser wavelength. The periodic nano-textures are the result of the spatial energy distribution. When the incident laser beam interferes with surface electromagnetic waves generated by the rough surface which induces the excitation of surface plasmon polaritons (SPP) [28,44,45], then the periodic ripples are generated.

**Figure 6:**
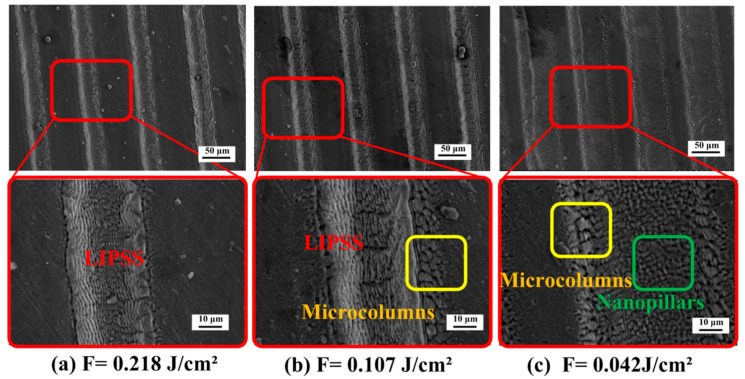
Effects of laser energy density on the morphology of LIPSS (scanning speed is 40 mm/s, the pulse frequency is 0.1 MHz, the single pulse energy is 75 μJ). The laser energy density is (**a**) F = 0.218 J/cm²; (**b**) F = 0.107 J/cm²; (**c**) F = 0.042 J/cm².

The preparation of the LIPSS structure is discussed in connection with scanning speed and energy density. When the scanning speed is kept at 40 mm/s, the energy density was changed by adjusting the amount of defocus and the laser spot diameter. The obtained surface structure is shown in Figure 6. In Figure 6a, it is observed that a significant LIPSS is generated on the scanning trajectory when the defocusing amount is 0.5 mm and the energy density is 0.218 J/cm^2^. The result is similar to the research of Kon et al. [46]. As the amount defocus increases to 1 mm, the energy density reduces to 0.107 J/cm^2^. In Figure 6b, the surface structure is composed of a periodic ripple structure and a microcolumns structure. However, the obtained structure is not distinct due to the decrease of laser energy density. Furthermore, when the defocusing amount increases to 3 mm, the energy density is 0.042 J/cm^2^. The microstructure of the scanning trajectory is composed of microcolumns and nanopillar structures, and no LIPSS is observed in Figure 6c.

**Figure 7:**
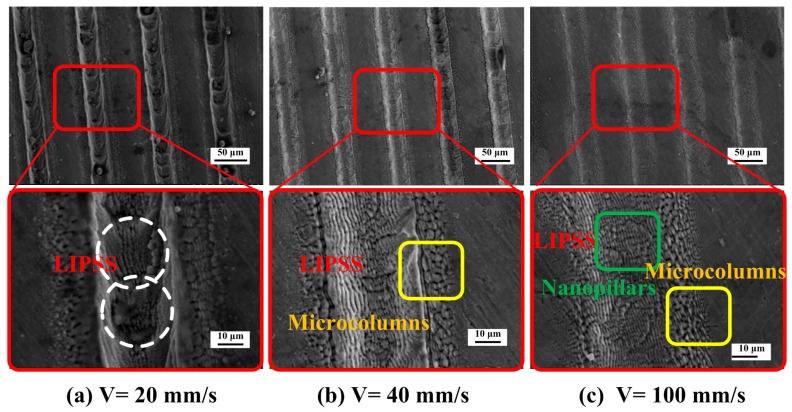
Effect of scanning speed on the morphology of LIPSS (defocusing amount L is 1 mm, laser energy density is 0.107 J/cm², the pulse frequency is 0.1 MHz, the single pulse energy is 75 μJ). The scanning speed is (**a**) V= 20 mm/s; (**b**) V= 40 mm/s; (**c**) V= 100 mm/s.

Figure 7 shows the effect of scanning speed on the morphology of the periodic structure. The defocus amount is controlled at 1 mm with the energy density of 0.107 J/cm^2^. Figure 7a shows the microstructure as the scanning speed is 20 mm/s. There are some splashing and redeposition of the molten material on the surface scanning trajectory. Its surrounding region and the obvious laser spot overlap trail can be seen from the trajectory in the partial enlarged view, which indicated by the white dashed box marked in the figure. As the scanning speed is slower and the number of pulses is larger, the cumulative energy is higher. Then, severe damage of LIPSS formed on the surface. Figure 7c shows the microstructure as the scanning speed is 40 mm/s. The periodic ripples are generated inside the scanning trajectory. The densely microcolumns are generated due to the lower energy density at the edge of the spot, which obeys the Gaussian distribution of the laser energy. Figure 7c shows the microstructure as the scanning speed is 100 mm/s. A small amount of periodic ripple structure and microcolumns structure exist inside and its edge of the scanning track. The obtained ripples are unclear, which means the surface cumulative energy density than the damage threshold of the material surface. Therefore, a clear and regular periodic ripple structure cannot be induced in the substrate surface.

When the laser energy density and the scanning speed are adjusted, a LIPSS structure with obvious structural features can be obtained on the surface of the substrate. Therefore, the LIPSS can be obtained on the surface of Ti-6Al-4V when the defocusing amount is 0.5~1 mm, the energy density is 0.107 J/cm^2^~0.218 J/cm^2^ and the scanning speed is 30~50 mm/s.

**Figure 8:**
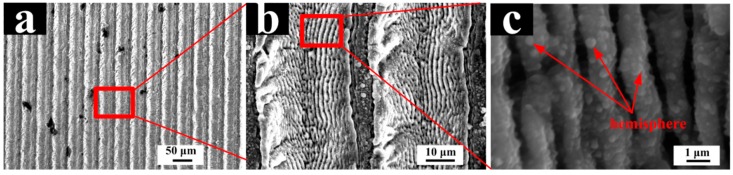
LIPSS induced on the Ti-6Al-4V surface with a laser energy density of 0.218 J/cm² and scanning speed of 40 mm/s. (**a**) Periodic ripples; (**b**) enlarged view, ×1500; (**c**) enlarged view, ×13,000.

Figure 8 shows the LIPSS structure has a period of about 1100 nm and the ripple structure is parallel to the laser polarization direction. In Figure 8c, a densely distributed hemispherical protrusion is formed on the periodic ripple structure with the diameter of 100–300 nm. The complicated surface structure has an advantageous effect on improving surface hydrophobicity.

#### 3.2.2. Preparation of Micro/Nano Hierarchical Structure

**Figure 9:**
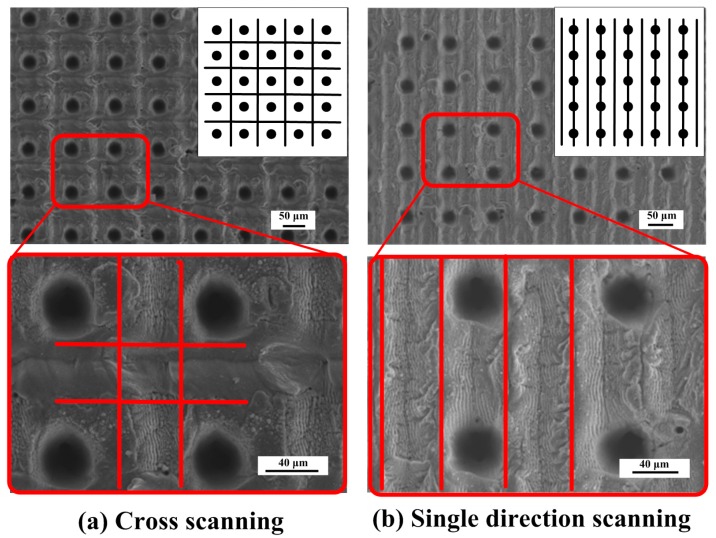
The FE-SEM image of the hierarchical structure with a micro-dimple array and LIPSS: (**a**) Cross scanning; (**b**) single direction scanning.

Finally, the micro/nano hierarchical structure is obtained on the Ti-6Al-4V surface by overlaying the periodic ripple structure on the prepared micro-dimple array structure without changing the laser source and the location of material. Figure 9 shows an SEM image of the hierarchical structure. The periodic ripple structure is distributed in the micro-dimple array and unablated regions uniformly, which exhibits a micro/nano hierarchical structure with typical characteristics. Moreover, different overlay ways were attempted to contrast the distinction between the obtained hierarchical structures. When the way of cross-scanning is adopted, the post-scan trajectory destroys the periodic structure formed by the first scan. Accordingly, the hierarchical structure (Figure 9b) prepared by the single direction lap method is more complete and clear. The complete overlapping of the scanning trajectory boundaries is achieved by adjusting the scan spacing and the spot size.

### 3.3. Wettability Property

The initial contact angle of the polished Ti-6Al-4V surface is 71.6°. Nevertheless, the contact angle of the laser textured surface shows a low contact angle that is less than 30° and all surfaces show the high hydrophilic property, and our results are similar with Li and Yangs’ [31,34]. The different phenomenon was observed when the samples stored in the air for some time. The CA of the stored sample increases with time and the surface changes from hydrophilicity to hydrophobicity finally. This transition process is as long as four weeks. However, the transition can be finished in a few hours after low-temperature annealing treatment.

**Figure 10:**
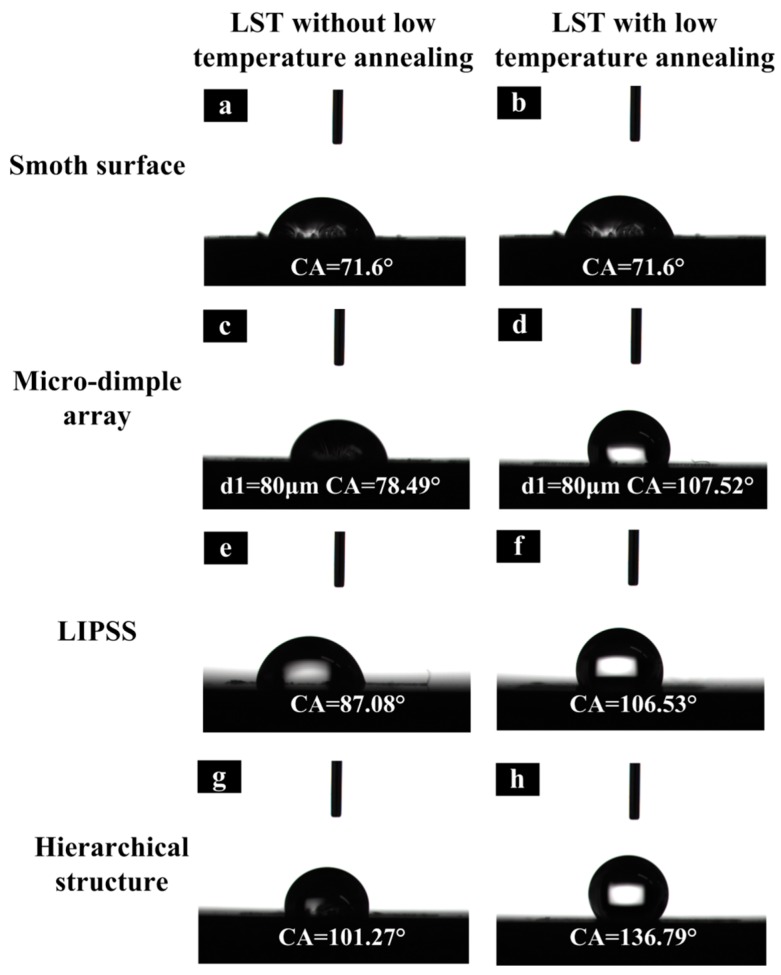
The contact state and contact angles of the textured surface: (**a**) untreated surface; (**c**) micro-dimple-array with a pitch distance of 80 μm and a diameter of 40 μm; (**e**) LIPSS texture; (**g**) the hierarchical texture; (**b**,**d**,**f**,**h**) represent the corresponding surface by low-temperature annealing treatment, respectively.

The relationship between the wettability of the textured surface and morphology of microstructure is investigated. The samples were divided into two groups, with or without the low-temperature annealing treatment. Figure 10 shows the contact state and contact angles of various laser textured surfaces with or without low-temperature annealing treatment. In Figure 10a,b, the contact angle of the smooth surface does not change after low-temperature annealing treatment. It indicates that the low-temperature annealing treatment has little effect on the wettability of the smooth surface. However, the contact angle of the textured surface treated by low-temperature annealing changed significantly. In Figure 10c,d, the contact angle of the surface with micro-dimple array increases from 78.49° to 107.52°. While the contact angle of the surface with LIPSS increases from 87.08° to 106.53° in Figure 10e,f. In Figure 10g,h, the contact angle increases from 101.27° to 136.79° when the surface of the micro-dimple array is covered by LIPSS. The contact angle reaches its maximum value.

### 3.4. Mechanism of Wettability Transition

#### 3.4.1. Effects of Nano-, Micro-, and Hierarchical Structure on the Contact Angle

In order to clarify the relationship between the structural parameters and the contact angle, the theoretical values of the contact angles in the Wenzel and Cassie states were calculated.

The calculation formula of the Wenzel model is,

(1) $$\cos\theta_{w}=r\;\cos\theta$$

where *θ*_w_ is the apparent contact angle of the Wenzel state. r is defined as the roughness, which is the ratio of the actual contact area to the apparent contact area (r ≥ 1). *θ* represents the contact angle of a smooth surface.

The calculation formulas of the Cassie–Baxter model are,

(2) $$\cos\theta_{c}=f_{1}\cos\theta_{1}+f_{2}\cos\theta_{2}$$

(3) $$f_{1}+f_{2}=1$$

where *θ*_c_ is the apparent contact angle, *f*_1_ is the surface fraction of the liquid–solid interface, and *θ*_1_ is the contact angle for the liquid–solid interface. *f*_2_ is the surface fraction of the liquid–vapor interface and *θ*_2_ is the contact angle for the liquid–vapor interface.

**Figure 11:**
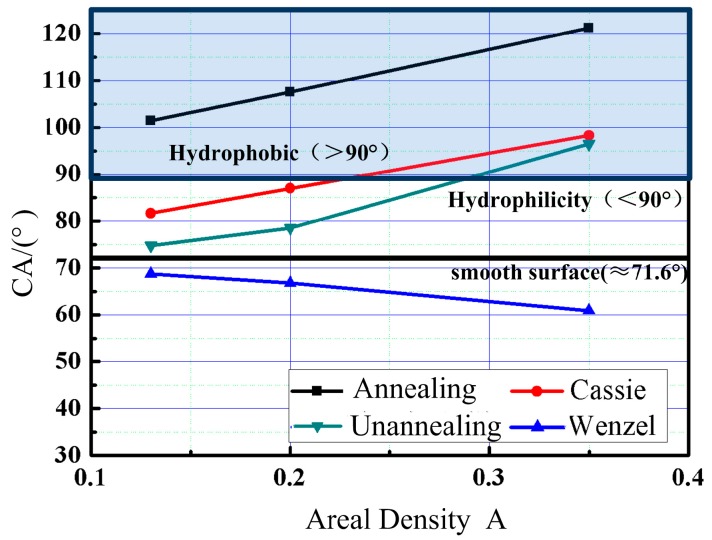
The relationship between the areal density of micro-dimple array and the CA.

The relationship between the measured results of the CA on the surface covered with the micro-dimple array and the surface density of the micro-dimple array is shown in Figure 11. The contact angle increases with the increase of the surface density of the micro-dimple array increases and has a consistent trend with the calculated value of the Cassie–Baxter model. Thereby, it can be considered that the wetting state of a deposited droplet on the textured surface in this experiment is approximated to Cassie–Baxter state.

Comparing the CA of textured surfaces with theoretical values of Cassie model, it is found that the CA of the unannealed textured samples is lower than the theoretical value. The CA of the annealed textured samples is significantly larger than the theoretical value, which is due to the difference of surface element composition and content of the samples. The elements affect the distribution of surface free energy, which leads to the error between experimental results and theoretical values.

It is assumed that the wetting state of the hierarchical surface satisfies the Cassie state. The calculation of theoretical values was carried out to verify the hypothesis based on Equation (4).

(4) $$\left\{ \begin{array}{l} \cos{\theta_{c}}^{\prime }={f_{1}}^{\prime }\cos\theta_{1}+{f_{2}}^{\prime }\cos\theta_{2}\\ {f_{1}}^{\prime }=f_{LIPSS}\cdot f_{1}\\ {f_{2}}^{\prime }=f_{2}+f_{LIPSS}\cdot f_{1} \end{array}\right.$$

where *θ*_c_′ is the apparent contact angle, *f*_1_′ and *f*_2_′ are the surface fraction of the liquid–solid interface and a surface fraction of liquid–vapor interface on the hierarchical surface, *f_LIPSS_* is the surface fraction of the LIPSS.

The hierarchical surface is composed of a micro-dimple array and the LIPSS. First of all, we hypothesize that the surface areal density of the LIPSS is 50% on account of its isometric ripple. When the surface areal density of the micro-dimple array varied, the apparent contact angle *θ_c_*′ is calculated by the equation set (4). When the surface areal density is 20%, the *θ_c_*′ is 118.28° while the experimental value is 136.79°. Meanwhile, when the surface areal density is 35%, the *θ_c_*′ is 124.92° while the experimental value is 144.58°. Then, it can be concluded that the wetting state of the hierarchical surface satisfies the Cassie–Baxter non-wetting state. Nevertheless, the results show that the experimental value is significantly larger than the theoretical value. The elemental composition of the treated samples has an obvious change. Then, the inherent surface free energy of the textured surface is modified, which leads to the surface wettability changing.

#### 3.4.2. Effects of Low-Temperature Annealing on the Contact Angle

It is noted that the surface microstructure does not change significantly whether the sample is exposed to air or treated by low-temperature annealing. Therefore, the surface wettability is mainly affected by the surface chemistry. The energy dispersive X-ray spectroscopy (EDS) was used to analyze and discuss the surface composition of all the samples.

**Figure 12:**
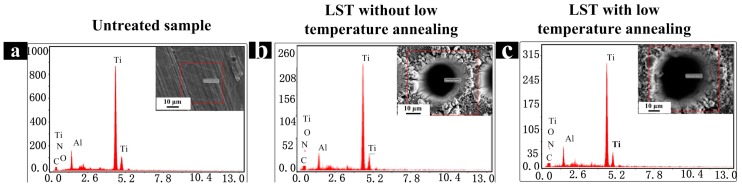
EDX energy spectrum of samples. (**a**) Untreated sample; (**b**) LST with low-temperature annealing; (**c**) LST without low-temperature annealing.

The test results of EDS for untreated sample and the surface with or without low-temperature annealing samples are shown in Figure 12 and Table 2. It is found that the mass ratio of surface O elements and the mass ratio of Ti increased from 3.41% to 40.88% and decreased from 81.7% to 50.6%, respectively. It can be concluded that the surface textured by laser occurred different degrees of oxidation phenomenon which result in the increase of the content of surface titanium oxide, especially TiO_2_ significantly. However, the mass fraction of Ti has almost no changes before and after annealing.

The greater the surface polarity is, the greater the surface energy is. The total surface free energy determines the surface wettability [29,47]. Titanium dioxide surface has a large number of polar sites, which makes the surface exhibit a high degree of non-equilibrium and surface polarity. Larger polarity can promote the dissociation and adsorption of ambient water vapor on the TiO_2_ surface, and then the hydroxyl groups are formed, which makes the fresh laser textured surface superhydrophilic [47,48]. After low-temperature annealing, a portion of the hydroxyl groups on the surface are removed [49,50], and other of the hydroxyl groups can chemisorb some non-polar organic chains from the air onto the laser textured surface [38,51,52]. Thus, the total surface energy is reduced and the surface becomes superhydrophobic. In addition, the texture has an amplification effect on the superhydrophobic surfaces [53]. Therefore, the laser textured surface after annealing has a large contact angle and is superhydrophobic.

## 4. Conclusions

In this paper, a 1064 nm pulsed picosecond laser was adopted to fabricate the Ti-6Al-4V titanium alloy texture surface with a micro/nano hierarchical structure. The wetting characteristics of the surfaces have been studied. Then, a low-temperature annealing treatment was carried out to accelerate the surface wettability transition. The mechanism of this transition was discussed. The following conclusions are drawn.

(1) Micro-dimple arrays with accurate size were achieved by adjusting the pulse energy and the number of pulses. In addition, the dimple array is covered with laser-induced periodic ripple structure (LIPSS). The ripples have a period of about 1100 nm when the energy density and scanning speed is 0.107 J/cm^2^~0.218 J/cm^2^ and 30~50 mm/s, respectively. Thus, the micro/nano hierarchical structure is obtained in the Ti-6Al-4V surface.
(2) The contact angle increases significantly with the increase of areal density. Surface wettability of micro and micro/nano hierarchical structure is consistent with the Cassie–Baxter state. At the same time, when the micro-dimple array surface is covered with the LIPSS (periodic ripple structure), contact angle values can reach the maximum value, 144.58°.
(3) The change of hydroxyl groups on the surface is the main cause of surface hydrophobicity. Low-temperature annealing treatment can accelerate the transition of wettability.

## Figures and Tables

**Table 1:** Parameters of laser for preparing micron/nanoscale textures.

a Micro-Dimple Array		b LIPSS	
Single-pulse energy	75 μJ	Single-pulse energy	75 μJ
Pulse frequency	400 kHz	Pulse frequency	100 kHz
Scanning speed	300 mm/s	Scanning speed	20 mm/s~100 mm/s
Defocusing amount	0~2.5 mm	Defocusing amount	0~4 mm
Pulse number	20~200	Scanning times	1
Energy density	6.68~16.7 J/cm^2^	Energy density	0.02~ 66.8 J/cm^2^
Accumulated fluence	334~1336 J/cm^2^		

Acc. Fluence = Fluence × Number of Pulses.

**Table 2:** Element composition of untreated sample and LST surface with or without low-temperature annealing (wt %).

Main Elements	C	N	O	Al	Ti
Untreated sample	1.24	3.7	3.41	6.34	81.7
LST without low temperature annealing	2.2	2.32	40.88	4.01	50.6
LST with low temperature annealing	3.11	0.09	42.6	3.85	50.35
